# Is 2D speckle tracking echocardiography useful for detecting and monitoring myocardial dysfunction in adult m.3243A>G carriers? — a retrospective pilot study

**DOI:** 10.1007/s10545-016-0001-7

**Published:** 2017-01-04

**Authors:** S. Koene, J. Timmermans, G. Weijers, P. de Laat, C. L. de Korte, J. A. M. Smeitink, M. C. H. Janssen, L. Kapusta

**Affiliations:** 1 Radboud Centre for Mitochondrial Medicine, Radboud University Nijmegen Medical Centre, Geert Grooteplein 10, 6500 HB PO BOX 9101, Nijmegen, The Netherlands; 2 Department of Cardiology, Radboudumc, Nijmegen, The Netherlands; 3 Clinical Physics Laboratory, Department of Radiology, Radboudumc, Nijmegen, The Netherlands; 4 Department of Internal Medicine, Radboudumc, Nijmegen, The Netherlands; 5 Department of Paediatrics, Paediatric Cardiology Unit, Tel-Aviv Sourasky Medical Centre, Tel Aviv, Israel; 6 Children’s Heart Center, Radboudumc, Amalia Children’s Hospital, Nijmegen, The Netherlands

## Abstract

**Objectives:**

Cardiomyopathy is a common complication of mitochondrial disorders, associated with increased mortality. Two dimensional speckle tracking echocardiography (2DSTE) can be used to quantify myocardial deformation. Here, we aimed to determine the usefulness of 2DSTE in detecting and monitoring subtle changes in myocardial dysfunction in carriers of the 3243A>G mutation in mitochondrial DNA.

**Methods:**

In this retrospective pilot study, 30 symptomatic and asymptomatic carriers of the mitochondrial 3243A>G mutation of whom two subsequent echocardiograms were available were included. We measured longitudinal, circumferential and radial strain using 2DSTE. Results were compared to published reference values.

**Results:**

Speckle tracking was feasible in 90 % of the patients for longitudinal strain. Circumferential and radial strain showed low face validity (low number of images with sufficient quality; suboptimal tracking) and were therefore rejected for further analysis. Global longitudinal strain showed good face validity, and was abnormal in 56–70 % (depending on reference values used) of the carriers (*n* = 27). Reproducibility was good (mean difference of 0.83 for inter- and 0.40 for intra-rater reproducibility; ICC 0.78 and 0.89, respectively). The difference between the first and the second measurement exceeded the measurement variance in 39 % of the cases (*n* = 23; feasibility of follow-up 77 %).

**Discussion:**

Even in data collected as part of clinical care, two-dimensional strain echocardiography seems a feasible method to detect and monitor subtle changes in longitudinal myocardial deformation in adult carriers of the mitochondrial 3243A>G mutation. Based on our data and the reported accuracy of global longitudinal strain in other studies, we suggest the use of global longitudinal strain in a prospective follow-up or intervention study.

**Electronic supplementary material:**

The online version of this article (doi:10.1007/s10545-016-0001-7) contains supplementary material, which is available to authorized users.

## Introduction

Cardiomyopathy is a common complication of mitochondrial disorders, with a prevalence up to 14 % in adults (Arpa et al. 2003) and 58 % in children (Holmgren et al. 2003; Scaglia et al. 2004). The presence of cardiomyopathy has been associated with increased mortality in both children and adults (Holmgren et al. 2003; Scaglia et al. 2004; Majamaa-Voltti et al. 2008; Malfatti et al. 2013). In carriers of the m.3243A>G mutation, one of the most common genetic causes of mitochondrial disease. Reference chinnery, the prevalence of either symptomatic or asymptomatic cardiomyopathy ranges from 18 to 56 %, depending on the population studied and the method used (Majamaa-Voltti et al. 2002; Vydt et al. 2007; Malfatti et al. 2013). Cardiomyopathy associated with this mutation was characterized by concentric left ventricle hypertrophy (Majamaa-Voltti et al. 2002; Bates et al. 2013a, b) with progressive dilation and decreased systolic function, developing over several years (Okajima et al. 1998). Some (but not all) of these small studies, reported a higher incidence of cardiac involvement in severe disease and patients with a high heteroplasmy percentage (Majamaa-Voltti et al. 2002; Vydt et al. 2007; Hollingsworth et al. 2012).

The m.3243A>G mutation is a mitochondrial DNA mutation and therefore follows maternal inheritance (Smeitink et al. 2006). To understand the importance of this study, the following aspects have to be considered: i) the mutation is present in a variable percentage of all mtDNA (heteroplasmy); ii) virtually all organs may be affected in various different patterns; and iii) this is not only dependent on the heteroplasmy percentage of the mutation.

The severity of the myocardial dysfunction is usually assessed by conventional echocardiography and tagged cardiac magnetic resonance imaging (cMRI). Previously, tagged cMRI showed abnormal myocardial deformation in 22 asymptomatic m.3243A>G carriers (Bates et al. 2013a, b). cMRI is used as a reference standard for myocardial deformation, but it is a time-consuming and expensive procedure which requires dedicated expertise. Therefore, this method is less feasible as bed side modality in multi-centre trials. Conventional echocardiography, in contrast, is widely available, but has low sensitivity in detecting subtle and regional changes in myocardial function (Aurigemma et al. 1995). The off-line processing of conventional echocardiograms using two dimensional speckle tracking echocardiography (2DSTE) software enables more sensitive quantification of the global and regional myocardial deformation (Artis et al. 2008). This software tracks myocardial ‘speckle’ patterns throughout the myocardial cycle, facilitating calculation of myocardial deformation or strain in three directions (longitudinal, radial and circumferential). The technique is reliable (Mavinkurve-Groothuis et al. 2009) and accurate (Amundsen et al. 2006; Choi et al. 2013) and is able to detect subtle changes in myocardial function at an early stage, even before decrease in conventional echocardiographic parameters (e.g. ejection fraction and shortening fraction) is observed (Poterucha et al. 2012). Recently, global longitudinal strain, known as the most reliable component of strain analysis (Kocabay et al. 2014), was incorporated into the recommendations for multimodality imaging evaluation and monitoring of cardiac (dys)function of adult patients during and after cancer therapy (Plana et al. 2014). A 10 % decrease in the longitudinal strain is a significant outcome measure for chemotherapy induced cardiomyopathy.

In this retrospective pilot study we evaluate the usefulness and feasibility of 2DSTE in detecting and monitoring subtle changes in myocardial deformation in adult carriers of the m.3243A>G mutation with a wide spectrum of clinical disease severity, since 2DSTE might be a potential outcome measure to evaluate responsiveness to future therapy for this disease.

## Methods

### Study population

All subjects were identified from our “National inventory of patients with the m.3243A>G mutation” study, including both symptomatic and asymptomatic carriers (de Laat et al. 2012). Subjects with a detectable heteroplasmy percentage (detection limit ≥5%) in either buccal mucosa cells, leukocytes, or urinary epithelial cells (UEC) are considered to be carriers of the mutation. As part of usual clinical care, both symptomatic and asymptomatic carriers undergo cardiac ultrasound (approximately two-yearly in asymptomatic individuals; more frequently if clinically indicated). Carriers of the m.3243A>G mutation of whom two subsequent echocardiograms were available were included in this study. Only part of these patients were included in our previously published study on biomarkers (Koene et al. 2015).

### Clinical assessment protocol

The clinical assessment at the time of the echocardiogram included the carrier’s medical history, current cardiac complaints and the use of medication, physical examination (including blood pressure, height and weight) and an electrocardiogram (ECG).

In the context of the “National inventory of patients with the m.3243A>G mutation”, patients and their maternal relatives undergo several investigations including the assessment of general mitochondrial disease severity using the Newcastle Mitochondrial Disease Adult Scale (NMDAS) (Phoenix et al. 2006). The NMDAS score closest in time to the echocardiography was reported. The NMDAS contains the following four domains: i) current function; ii) system specific involvement; iii) current clinical assessment; and iv) quality of life. In our analysis, we used domains i-iii to calculate disease severity. Severe mitochondrial disease was defined previously as an NMDAS score above 20 (de Laat et al. 2012). We defined asymptomatic disease as an NMDAS = 0; mild mitochondrial disease as an NMDAS score of 1 through 5 and moderate mitochondrial disease as an NMDAS score of 6 through 20 (Koene et al. 2014). The presence and severity of diabetes mellitus (DM) and cardiovascular involvement was also obtained from the NMDAS (Schaefer et al. 2006). Quality of life, with sub-scores for mental and physical quality of life, was determined using a Dutch translation of the SF-12v2 and American reference values SFv12 ref. The quality of life can vary from 0 to 70 for both mental and physical health, where 50 is the population’s mean (standard deviation 10). Subjective change during follow-up was part of the general history taking of the cardiologist.

Laboratory investigations (+/− 6 months) were reported. Carriers were classified as having decreased creatinine clearance only, microalbuminuria only, both or neither. Microalbuminuria was defined as an albumin-to-creatine-ratio of >2.0 g/mol for men and >2.5 g/mol for women. Decreased creatinine clearance was defined as a glomerular filtration rate <60 ml/min/1.73 m^2^.

### Echocardiography

All carriers underwent a transthoracic 2D echocardiogram in supine and lateral position at rest. The echocardiogram was performed as part of our regular patient care, using a standardized echocardiographic protocol published previously (Bulten et al.^2014^), by an experienced echocardiography technician and supervised by experienced cardiologist (JT). Images were obtained with an M3S transducer using the Vivid 7 and M5S transducer using Vivid E9 echographic scanners (GE, Vingmed Ultrasound, Horten, Norway). Quantification of cardiac chamber size, left ventricular mass and systolic and diastolic left ventricular function were performed in accordance with the recommendations for chamber quantification by the American Society of Echocardiography’s Guidelines and Standard Committee and the Chamber Quantification Writing Group (Lang et al. 2005; Nagueh et al. 2009), as previously described (Bulten et al. 2014). In case the ejection fraction (EF) was not available, fractional shortening (FS) was used.

**Fig. 1:**
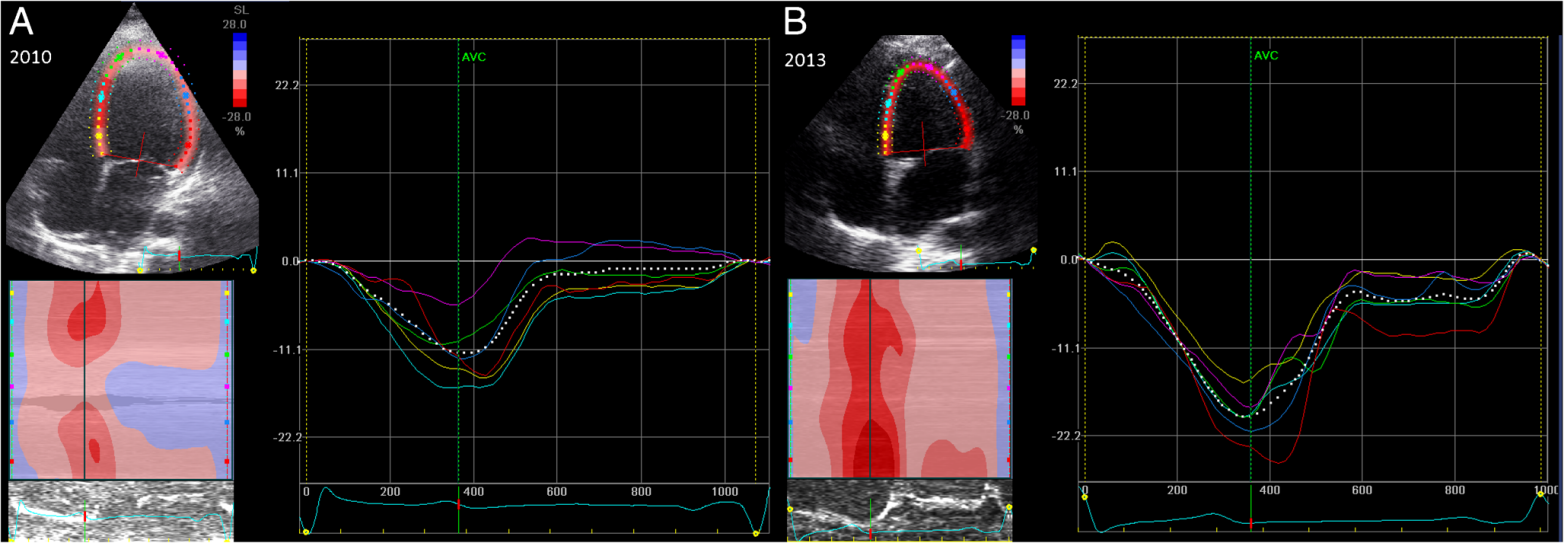
Two-dimensional strain measurement. Two-dimensional strain measurement in longitudinal plane. At the left top, the region of interest is shown, at the right the graphic depicture of the transmural strain in a young female m.3243A>G carrier who was found to have severe cardiomyopathy in 2010. After diagnosis, she was started on medication (diuretics, ACE inhibitor and β blocker) and underwent an intensive heart failure rehabilitation programme. At the echocardiogram in 2013, she reported a highly significant increase in exercise tolerance. The coloured lines represent the measurements of regional deformation of the individual regions, the dotted line represents their average (global strain), analysed by GE EchoPac. GLS increased from −12.7 to −19.6

Strain analysis was done according to our previously published protocol (Mavinkurve-Groothuis et al. 2009) by an experienced investigator (LK), using two-dimensional grey scale images taken in the parasternal apical 4-chamber view (4-CV) and at mid-cavity short-axis view (at the level of the papillary muscle; SAX-PM). The investigator was not aware of the clinical condition and medical treatment of the patients. A sector scan angle of 30–60° was chosen and frame rates of 70 Hz or more were used (Leitman et al. 2004). Cine loops of preferably three cardiac cycles triggered by the R wave of the QRS complex were digitally saved. Offline analysis was performed using software for echocardiographic quantification (EchoPAC 6.1.0, GE Medical Systems, Horten, Norway). Timing of aortic valve closure and mitral valve opening was used to indicate end-systole and start of diastole respectively. Manual tracking of the endomyocardial borders was performed at the end-systolic frame. An automatic generation of the second epicardial tracing was created by the software, which also automatically divided the LV myocardium into six equal segments, which were named and localized according to the statement of the Cardiac Imaging Committee of the Council on Clinical Cardiology of the American Heart Association (Cerqueira et al. 2002; Voigt et al. 2015). Quality of the speckle tracking was verified for each segment and adjusted when needed. Tracking was only accepted if visual inspection indicated adequate tracking over the full cardiac cycle. Preferably, three cardiac cycles were analysed for each segment and exported as text files for further post-processing. As a next step the exported strain curves were post-processed, in a custom made software package using Matlab (r2013b), in order to estimate the average strain curve and to obtain the final strain parameters out of this data (Mavinkurve-Groothuis et al. 2009). Figure 1 shows a composite figure with the region of interest (left side) and graphic depiction of longitudinal strain in 4-chamber long axis view.

To evaluate the conventional echocardiographic values obtained in our patient group, we used the reference values described by the American Society of Echocardiography (Lang et al. 2005; Nagueh et al. 2009). Strain values are dimensionless and are expressed in percentages (Berne and Levy 1993). Global longitudinal myocardial strain (GLS) was calculated by averaging the six segments of three cardiac cycles using the apical 4-chamber view (4-CV). Global radial and circumferential myocardial strain (GRS and GCS, respectively) were calculated by averaging the six of three cardiac cycles segments of the mid-cavity short-axis view (SAX-PM). When less than four segments were available for averaging, global strain was not calculated. Age-matched reference values for end-systolic strain were obtained from Kocabay et al. (Kocabay et al. 2014) and from Kuznetsova et al. (Kuznetsova et al. 2008). Values were considered abnormal when deviating >2 standard deviations (SD) from mean (Kocabay) or < *P*5 or > *P*95 (Kuznetsova, SDs were not reported). Our group (LK, CdK, GW) has published multiple studies on myocardial strain in both children and adults (Marcus et al. 2011; Mavinkurve-Groothuis et al. 2013; Bulten et al. 2014). A recent meta-analysis shows that the LV GLS reference values for children obtained by our group are well within the averaged 95 % CI (Levy et al. 2015). Each carrier was compared individually to his/her age-matched reference values. Changes were reported as absolute numbers and changes of ≥ 10 % were accepted as real changes, as this number exceeds the coefficient of variation reported in literature (Mele et al. 2015) recommended by Plana et al.

### Reproducibility studies

Ten images in which strain analysis was possible were randomly selected by a physician not involved in the strain measurements for reproducibility studies. To determine inter-observer reproducibility, longitudinal strain was analysed by another experienced rater (GW), blinded to previous results. Intra-rater reproducibility was determined by rating the same images, more than 6 months later. For both inter- and intra-observer reproducibility, absolute differences and the intraclass correlation coefficients were calculated.

### Follow-up study

Only carriers of whom two echocardiograms in which GLS analysis was feasible were available and were included in the follow-up study. Subjective and objective changes in clinical status, changes in the use of medication and changes in conventional parameters were assessed during follow-up. Since all echocardiograms were performed as part of clinical care, the time between two examinations is variable.

### Medical ethical approval

This study is part of the “National Inventory of Patients with the m.3243A>G mutation”, which was approved by the regional Medical Research Ethics Committee. In accordance with the Helsinki agreement, written informed consent was obtained from each participant.

### Statistical analysis

The absolute difference was calculated by subtracting the first measurement from the second measurement. All variables were assessed for (log)normality. To prevent non-real values for zero values of the NMDAS including its sub domains and symptom specific items, these values were increased by 1 prior to ^e^log transformation. Variables with a (log)normal distribution, were compared using parametric tests, and the mean and 95 %-confidence intervals (95 % CIs) are reported. Variables that deviated strongly from a (log)normal distribution (based on skewness/kurtosis) were analysed by performing a non-parametric test and the median and interquartile ranges (IQRs) are reported. Inter- and intra-rater reliability were calculated using intraclass correlation coefficients for absolute agreement (ICC). Outliers were not excluded from any of the analyses and missing data were not replaced. In case a high number of tests were performed (5 or more), critical *P*-values were adjusted using the Bonferroni method (i.e. 0.05/*n* where *n* = number of tests). Correlation coefficients were interpreted in accordance with the guidelines provided at the BMJ website (http://www.bmj.com/about-bmj/resources-readers/publications/statistics-square-one/11-correlation-and-regression; consulted 29-June-2015).

All analyses were performed using IBM’s SPSS statistics software packages, version 20.0.0.1.

## Results

### Carrier description

**Fig. 2:**
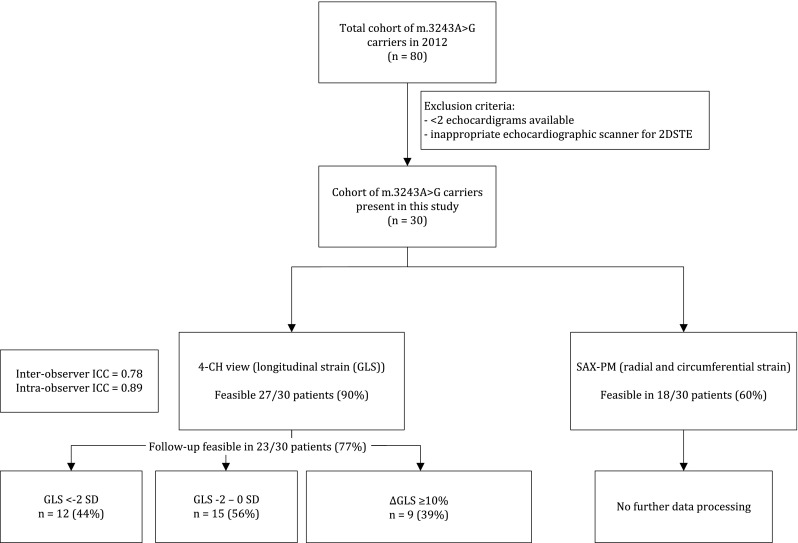
Study algorithm

The study algorithm is presented in Fig. 2. Thirty carriers of the m.3243A>G mutation of whom two subsequent echocardiograms were available for 2D strain analysis were included in this study (Table 1). Nineteen of these carriers were female; five of them were current smokers. Eighteen carriers had diabetes mellitus, six microalbuminuria, one decreased creatinine clearance and ten carriers had cardiovascular involvement according to the NMDAS.

**Table 1:** Carrier characteristics. Clinical features of the included carriers compared to all adult carriers in the “National inventory of carriers with the m.3243A>G mutation”. The presence of diabetes mellitus was obtained from the NMDAS scale (score on diabetes mellitus item ≥3); the presence of cardiovascular involvement was obtained from the NMDAS scale (score on cardiomyopathy ≥ 1). *P*-values (significance (*P* < 0.0045)) for the difference at baseline between this cohort and all adult carriers included before 2012 were calculated

		*n* =	Mean (range)		Total number of patients	Difference from total cohort of carriers (*n* = 80) *p* =
Gender	Female	19			30	
Age			45.2	(16.7–64.5)	30	0.90
BMI	(kg/m2)		23.8	(17.3–34.0)	30	0.006
Alcohol	Use	6			27	
	Abuse	1			27	
Smoking	Current	5			27	
	Stopped	5			27	
Renal abnormalities	Micro-albuminuria	6			30	
	Decreased creatinine clearance	1			30	
Diabetes mellitus (NMDAS)	Yes	18			30	0.37
HbA1c	(mmol/mol)		6.7	(5–9.7)	28	
Cardiovascular involvement (NMDAS)	Yes	10			30	0.84
Systolic blood pressure			127	(95–167)	23	
	<120 mmgHg	5				
	120–140 mmHg	13				
	>140 mmHg	5				
Diastolic blood pressure	(mmHg)		79	(58–98)	23	
Total cholesterol	(mmol/l)		4.8^a^	(3.5–9.2)	23	
HDL cholesterol	(mmol/l)		1.1	(0.6–1.9)	21	
LDL cholesterol	(mmol/l)		2.9	(0.9–4.8)	21	
Heteroplasmy in leucocytes	(%)		28	(3–73)	30	
Heteroplasmy in UEC	(%)		52	(7–96)	30	
NMDAS			18.6	(0–49)	30	0.35
Domain 1			7.5	(0–21)	30	0.11
Domain 2			7.3	(0–18)	30	0.20
Domain 3			2.6^b^	(0–13)	30	0.96
QoL mental			46	(25–59)	28	0.34
QoL physical			40	(21–58)	28	0.09
Medication^c^	β blocker	6			30	
	Calcium channel blocker	1			30	
	ACE inhibitor	6			30	
	Angiotensin II blocker	2			30	
	Insulin	5			30	

*BMI* body mass index; *domain 1* current function; *domain 2* system specific involvement; *domain 3* current clinical assessment; *IQR* interquartile range; *n* number of carriers of which data were available at that specific time point; *NMDAS* newcastle mitochondrial disease adult scale; *QoL* quality of life; *UEC* urinary epithelial cells

^a^median is given instead of mean

^b^lognormal distribution

^c^not mutually exclusive

In the “National Inventory of Patients with the m.3243A>G mutation”, 80 adult carriers had been included before 2012 (since the regular follow-up time is 2 years, we do not account for carriers included before 2012). For a variety of reasons (e.g. only one echocardiogram available or echocardiogram made using a wrong echocardiograph device), only data of 30 carriers (38 %) from 20 families could be analysed (1–3 family members per family). These 30 carriers were not different compared to the total cohort with respect to their total NMDAS score (*P* = 0.35), nor in their sub scores for diabetes mellitus or cardiovascular disease (*P* = 0.37 and 0.81 respectively; independent samples *t*-test). BMI was significantly higher in the included carriers compared to the other carriers (*P* = 0.0062).

### Conventional echocardiography at baseline

Table 2 presents the echocardiographic parameters of our patients. Although the measurement of cardiac chamber size, left ventricular mass and systolic and diastolic left ventricular function was feasible in 80–100 % of the patients, measuring EF was only feasible in 33 % of the patients. Using conventional echocardiography, seven carriers (23 %) did not present with (sub-clinical) cardiac abnormalities. Hypertrophy was reported in 40 % of the carriers, mild mitral insufficiency in 13 % and mild aortic insufficiency in 27 % of the carriers.

**Table 2:** Cardiac characteristics at baseline. Conventional echocardiographic and myocardial deformation in m.3243A>G carriers compared to the reference population. Reference values were obtained from Nagueh et al. and Lang et al. for the conventional echocardiographic parameters and from Kocabay et al. and Kuznetsova et al. for strain data. High (>+2SD) and low (−2SD) are based on age-matched reference values

	Median (spread)	High values (*n* (%))	Low values (*n* (%))	*n* =
FS (%)	36 (11–56)	2 (7 %)	10 (33 %)	30
EF (%)	64 (25–68)	–	3 (30 %)	10
Interventricular septum thickness in diatsole (cm)	0.9 (0.6–3.0)	12 (41 %)	–	29
LV posterior wall thickness (cm)	0.9 (0.7–1.4)	13 (45 %)	–	29
LV internal diameter in diastole (cm)	4.4 (1.9–6.0)	1 (3 %)	6 (21 %)	29
Mitral valve E/A ratio	1.1 (0.7–4.0)	2 (7 %)	1 (3 %)	29
LV performance (Tei) index	0.4 (0.3–0.8)	11 (41 %)	–	27
Isovolumic relaxation time (ms)	84 (50–120)	9 (32 %)	2 (7 %)	28
Pulmonary vein S/D ratio	1.4 (0.54–2.2)	5 (21 %)	–	24
Left ventricular mass index (g/m2)	76 (23–140)	4 (17 %)	2 (8 %)	24
Global longitudinal strain compared to Kocabay et al.	−16.3 (−7.9–−20.7)	−	15 (56 %)	27
compared to Kuzenetsova et al.		−	19 (70 %)	27

*E/A ratio* ratio between early (E) and late (A) filling velocity, measured at the mitral valve; *LV* left ventricular; *S/D ratio* ratio between the velocity of the flow in systole and diastole, ¤ not mutually exclusive

We found no statistically significant correlation between left ventricular mass index and the NMDAS (ρ(NMDAS) = 0.01, *P* = 0.97; *n* = 24) and heteroplasmy percentages (ρ(heteroplasmy UEC) = −0.01; *P* = 0.97; ρ(heteroplasmy leucocytes) −0.04, *P* = 0.87; *n* = 24).

### Strain feasibility

For the analysis of GLS on the 4-CV images, three patients (10 %) had to be excluded because no GLS analysis could be performed. Two instead of three cycles were suitable for analysis in 33 % (e.g. bad quality of the images, only two cardiac cycles recorded). The assessment of GLS was feasible in 151 (94 %) of the 161 segments available for analysis. During follow-up; two subsequent images could be analysed in 23 patients (feasibility 77 %).

For the SAX-PM view, 18 patients (30 %) had to be excluded because of low image quality. Only 57 % of the segments had sufficient image quality to perform radial and circumferential strain analyses. Because of the low number of high-quality images, the suboptimal tracking performance of some specific segments (mainly posterior and lateral) even in the high-quality images, and the current debate on the reproducibility of radial strain, (Koopman et al. 2010) we chose not to further process these results and concentrate only on the longitudinal 2D strain.

### Longitudinal 2D strain at baseline

When comparing our results to the age-matched reference values of Kocabay et al., 15 carriers (56 %) had abnormal GLS (Table 3). When comparing to the age-matched reference values of Kuznetsova, 19 carriers (70 %) had abnormal GLS. Of the seven carriers with normal cardiac function and diameters assessed by conventional echocardiography, two or four (depending on the reference values used) had abnormal GLS. In the ten carriers with low fractional shortening, five had low GLS (one GLS not available). In the nine carriers with a high diastolic left ventricular posterior wall thickness, seven had decreased GLS.

**Table 3:**
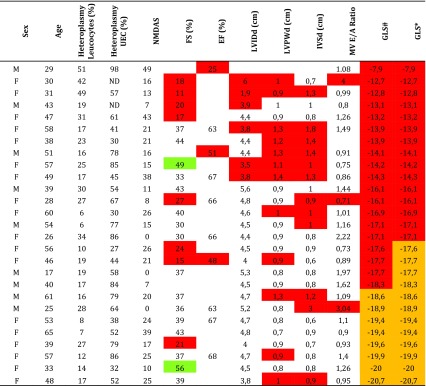
Global strain in carriers individually compared to age-matched controls. Global longitudinal end-systolic strain in m.3243A>G compared to the age-matched reference population (*n* = 27). tThe low values (under −2 SD for age-matched reference values) are marked in red, the values below the mean (between 0 and −2 SD) are marked in orange. The high values (above +2 SD) are marked in green

*E/A ratio* ratio between early (E) and late (A) filling velocity, measured at the mitral valve; *F* female; *FS* fractional shortening; *GLS* global longitudinal end-systolic strain; *GRS* global radial end-systolic strain; *LVPWd* diastolic left ventricular posterior wall thickness; *M* male; *NMDAS* newcastle mitochondrial disease adult scale; *UEC* urinary epithelial cells; * Kocabay reference values (95%CI); # Kuznetsova reference values (90 % CI)

No significant correlation between age and GLS was found (*r* = −0.21; *P* = 0.30). GLS was not significantly lower in carriers with DM (*P* = 0.68). GLS was not significantly lower in carriers with a score 0 versus ≥1 on the cardiovascular involvement item in the NMDAS (*P* = 0.10). There was no correlation between IVSd and GLS (*ρ* = 0.35, (*P* = 0.08). Carriers with renal abnormalities (including microalbuminuria) did not have significantly lower GLS compared to carriers without renal abnormalities (*P* = 0.65).

**Fig. 3:**
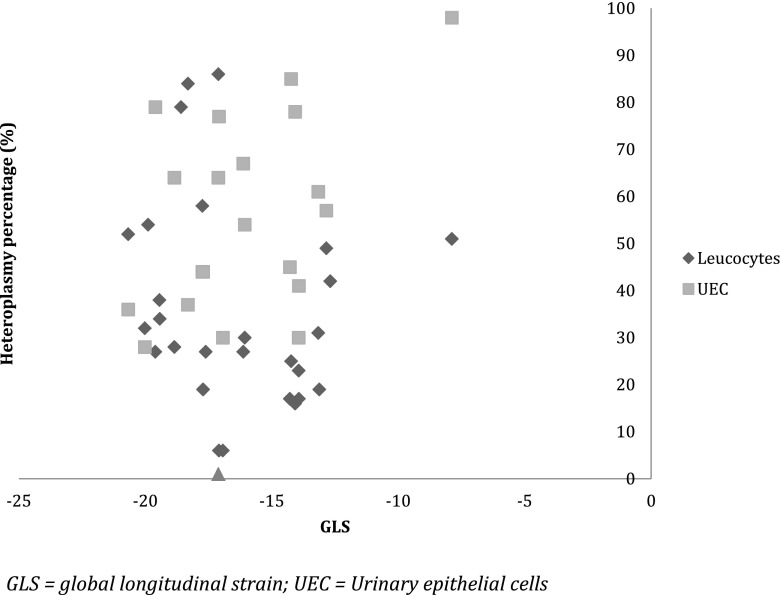
Correlation between heteroplasmy percentages and GLS. GLS correlates significantly to the heteroplasmy percentage in UEC *r* = 0.45, *P* = 0.05, but not to heteroplasmy percentage in leucocytes (*r* = −0.17, *P* = 0.36). GLS = global longitudinal strain; UEC = Urinary epithelial cells

GLS correlated significantly to the heteroplasmy percentage in UEC, but not to heteroplasmy percentage in leucocytes (*r* = 0.45, *P* = 0.05 and *r* = −0.17, *P* = 0.36, respectively; Fig. 3). No significant correlation between the NMDAS score and GLS was found (*r* = 0.29, *P* = 0.17). The score in the first, subjective domain of the NMDAS did not correlate significantly to GLS (*r* = 0.18, *P* = 0.41). There was no significant difference in the GLS between carriers with asymptomatic, mild, moderate and severe general mitochondrial disease severity (*P* = 0.65). Physical quality of life did not correlate significantly to GLS (*r* = 0.18, *P* = 0.38). No significant correlation between mental quality of life and GLS was found (*r* = 0.15, *P* = 0.49).

### Inter- and intrarater reliability

The ICC of inter-rater reliability was 0.78, with a mean difference of 0.83 (95 % CI 0.38–1.85). The ICC of intra-rater reliability was 0.89, with a mean difference of 0.40 (95 % CI 0.11–1.42).

### Follow-up

The (changes in) conventional echocardiographic parameters and GLS for each individual patient are shown in Supplementary Table 1. Twenty-three patients were suitable for the analysis of changes in myocardial deformation during follow-up (feasibility 77 %). Table 4 shows the changes in GLS, the subjective changes in exercise tolerance and changes in medications. The median time between the first and the second echocardiogram was 2.0 years (IQR 1.1–2.7 years; range 0.5–4.6 years). The disease severity (including the cardiovascular and diabetes mellitus score and sub domains) as well as the quality of life did not change significantly during follow-up (*P* = 0.11–0.92).

**Table 4:**
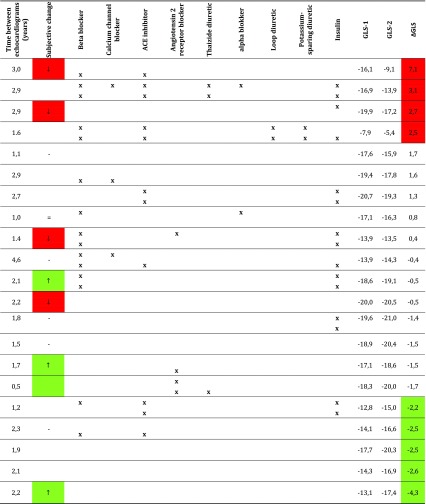
Clinical and strain parameters during follow-up in m.3243A>G carriers. Description of the change during follow-up and the change in global end-systolic strain in longitudinal direction at baseline and during follow-up in m.3243A>G carriers (*n* = 21). Time between echocardiograms, subjective change in exercise tolerance and changes in medication are also depicted. Red marking indicates a ≥ 10 % increase in myocardial strain; green marks a ≥ 10 % decrease in myocardial strain during follow-up

*GLS* global longitudinal end-systolic strain

Nine carriers (39 %) had a change in GLS ≥10 % at end systole. The change in GLS ranged from −7.1 to +6.9 (mean 0.33; 95%CI −5.5–+2.3), where a higher (thus positive) value represents an improvement in longitudinal strain.

Examples of the difference in responsiveness of myocardial strain include two cases of young carriers with m.3243A>G related cardiomyopathy which were documented in more detail: one young woman with a generally mild phenotype and newly discovered severe heart failure at the first echocardiogram recovered very well under pharmacotherapy and rehabilitation (GLS −12.7 to −19.6), while a male carrier with MELAS syndrome had a stable, severe heart failure (GLS −7.9 to −5.4). See Fig. 1 for the first case.

## Discussion

The aim of this retrospective pilot study was to evaluate the value and feasibility of 2DSTE in detecting and monitoring myocardial dysfunction in both symptomatic and asymptomatic carriers of the m.3243A>G mutation. Even when data collected as part of clinical care are used, 2DSTE seems a promising method to reliably quantify the abnormal longitudinal myocardial deformation (strain) observed in many m.3243A>G carriers with a wide spectrum of clinical disease severity. For this reason, it could be considered as an outcome measure in future clinical trials. Strain analysis was feasible in 90 % of the patients and 94 % of the images for global longitudinal strain (GLS). Radial and circumferential strain were not further processed because of a low number of high-quality images and suboptimal tracking even in high-quality images, which is in line with the current debate on the reproducibility of radial strain (Koopman et al. 2010). Decreased GLS was found in more than half of the m.3243A>G carriers; none of the carriers had a higher GLS than the average of the age-matched reference group. In most of the carriers with abnormal strain, cardiac dimensions were also abnormal, whereas the systolic function was still intact (Supplementary Table 1). Inter- and intra-rater reliability was good, with a mean difference of 0.83 for inter and 0.40 for intra-rater reproducibility. Since our centre has no experience in tagged cMRI, we could not confirm the very strong correlation between cMRI and 2DSTE for this specific indication.

A previous study showed that myocardial deformation, measured by cMRI, in m.3243A>G mutation carriers without known clinical cardiac involvement showed abnormal longitudinal shortening, whereas radial and circumferential strain were comparable to matched healthy controls (Bates et al. 2013a, b). This is in line with studies in other populations where GLS by echocardiography is reported to be the most sensitive strain parameter to assess systolic dysfunction (Nakai et al. 2009; Nesbitt et al. 2009). Global longitudinal strain is therefore accepted as a major outcome parameter of, e.g. chemotherapy related cardiac dysfunction (Plana et al. 2014). In general, GLS is reported to be relatively easy to measure and more consistent and reproducible compared to GCS (Kocabay et al. 2014). The hypothesis is that GLS is affected at first because the longitudinally orientated sub-endocardial fibres are most susceptible to injury (Nesbitt et al. 2009). In our present study, we found no correlation between the longitudinal myocardial deformation and the clinical parameters or clinical scoring such as the NMDAS. This might be attributed to the relatively small cohort, including both symptomatic and asymptomatic carries and the heterogeneous distribution of heteroplasmy percentages between tissues. Nevertheless, one should keep in mind that mild mitochondrial disease may be associated with severe cardiomyopathy and therefore all carriers of the m.3243A>G mutation should be screened for any sign of (subclinical) cardiomyopathy.

In another follow-up study of m.3243A>G carriers, cMRI was used to monitor cardiac adaptations and safety of endurance training. No difference was found in GCS during follow-up; however, GLS was not assessed (Bates et al. 2013a, b). Other studies report good responsiveness of radial (and in lesser extent of longitudinal) strain (Weidemann et al. 2003), and improvement of longitudinal strain under treatment (Faber et al. 2011) while others report no changes in myocardial strain during the follow-up of patients with progressive (not mitochondrial) diseases (St John Sutton et al. 2014). In the present pilot study, we found changes in GLS exceeding the inter- and intra-observer variability in 39 % of our cohort. Since we were not able to measure the disease progression with other methods than 2DSTE itself, one can not rule out that these changes in GLS could still partly represent the influence of other factors, e.g. changes in medication, or treatment of the most common cause of cardiac hypertrophy: hypertension. The influence of covariates of diminished myocardial deformation, including the decreased myocardial functioning associated with physiological aging (Cheng et al. 2010) and with the presence of diabetes mellitus (Ernande et al. 2010), was not significant in our cohort. This is probably caused by the high number of young subjects with severe m.3243A>G associated cardiomyopathy included in our cohort and the lack of correlation between the presence of m.3243A>G associated cardiomyopathy and m.3243A>G associated symptoms in general (including m.3243A>G associated diabetes mellitus). Although we found a moderate correlation between GLS and the heteroplasmy percentage measured in urinary epithelial cells, the heteroplasmy percentages in these tissues do not always represent the heteroplasmy percentage measured in cardiac muscle, since the m.3243A>G mutation is distributed unevenly across tissues (Majamaa-Voltti et al. 2008). Since all studies performed so far are quite small, larger studies need to be executed to be more conclusive about the discrepancy between our findings and other observations in small cohorts of mitochondrial patients (Bates et al. 2013a, b). These studies should spare no effort to measure systolic function, to confirm that abnormal strain indeed precedes abnormal ejection fraction and is therefore an earlier sign of myocardial dysfunction. These studies should also study the value of 2DSTE in risk stratification or risk prediction of cardiomyopathy in m.3243A>G carriers.

The analysis of myocardial deformation using tagged cMRI is considered the reference standard for measuring myocardial deformation (Tee et al. 2013), yet the lack of availability and expertise in many centres hampers its use in future clinical trials. Although 2DSTE is dependent on operator experience, machine settings and the acoustic window for transducer placement of the patient (Tee et al. 2013), the technique is recently often used in multi-centre clinical trials. Pitfalls of the 2DSTE method itself include its dependency on high quality images with sufficient frame rate and the limited standardization between vendors. Weaknesses of this pilot study include the data gathering as part of clinical care, the relatively small number of carriers of whom two echocardiograms were available for further 2DSTE, and the lack of standardized follow-up protocol to allow firm conclusions about the responsiveness of 2DSTE. Despite the fact that the 2D images were obtained as part of clinical care, the proportion of high quality images on which analysis was feasible was comparable to the literature (Stoodley et al. 2011).

More and more trials are performed in patients with mitochondrial disease. There is an urgent need for robust outcome measures (Pfeffer et al. 2013). The wide availability, the non-invasive nature and independence of voluntary effort of 2DSTE makes it promising as an objective and quantitative end point for clinical trials. The data for this retrospective pilot study were collected as part of routine care (using a standardized imaging protocol) and only 77 % of the patients were suitable for the assessment of longitudinal myocardial strain using 2DSTE. An important limitation of our study is that ejection fraction could only be calculated in a small number of patients. The discrepancy between the feasibility of the calculation of ejection fraction and the calculation of global longitudinal strain might be explained by the different images used for both techniques (4–6 segments of the 4-CV (2DSTE) versus all segments of both the 2-CV and the 4-CV (EF)), and by the fact that both parameters were assessed by different experts teams. Since these numbers are probably higher in highly standardized prospective studies, we recommend testing the feasibility and responsiveness of GLS in more detail in a prospective study. Myocardial deformation reflects not only the myocardial contractility, but probably also global forces such as pre- and afterload (Nesbitt et al. 2009). For proper use in future clinical trials, we suggest standardization of, e.g. medications and fluid intake at the time of the echocardiography.

Given the high prevalence of cardiac abnormalities and the lack of clear clinical predictors for the presence of not-yet-symptomatic cardiomyopathy, screening for this condition might be of major importance. Whether early detection of myocardial dysfunction in these patients will be beneficial for the long term prognosis in this population, remains to be further clarified. Although myocardial deformation seems to be predictive of heart failure (Choi et al. 2013) and all-cause mortality (Kalam et al. 2014), the value of treating subtle subclinical changes in myocardial deformation, detected using 2DSTE, should be studied prospectively in a larger cohort of adult m.3243A>G carriers.

## Electronic supplementary material

Below is the link to the electronic supplementary material.

Supplementary Table 1 Conventional echocardiographic parameters and GLS during follow-up for each individual m.3243A≥G carrier. Description change in conventional echocardiographic parameters as well as global end-systolic strain in longitudinal direction at baseline and during follow-up for all m.3243A>G carriers (*n* = 30). GLS = global longitudinal end-systolic strain (DOCX 31 kb)

## Abbreviations

2DSTE: 2-dimensional speckle tracking echocardiography
4-CV: 4 chamber view
95%CI: 95 % confidence interval
cMRI: Cardiac magnetic resonance imaging
DM: Diabetes mellitus
EF: Ejection fraction
FS: Fractional shortening
GLS: Global longitudinal strain
IQR: Inter quartile range
NMDAS: Newcastle mitochondrial disease adults score
SAX-PM: Short axis view at papillary muscle
SD: Standard deviation
UEC: Urinary epithelial cells
