# Supplementary material for: Is 2D speckle tracking echocardiography useful for detecting and monitoring myocardial dysfunction in adult m.3243A>G carriers? — a retrospective pilot study
Source: J Inherit Metab Dis. 2017 Jan 4;40(2):247–59. doi: 10.1007/s10545-016-0001-7 (PMC5306433; doi:10.1007/s10545-016-0001-7)
Supplement: Supplementary file 1 — Conventional echocardiographic parameters and GLS during follow-up for each individual m.3243A≥G carrier. Description change in conventional echocardiographic parameters as well as global end-systolic strain in longitudinal direction at baseline and during follow-up for all m.3243A>G carriers (n = 30). GLS = global longitudinal end-systolic strain (DOCX 31 kb) [file 10545_2016_1_MOESM1_ESM.docx]

Supp table 1

| **FS (%)** | | | **EF (%)** | | | | **Left ventricular mass index (g/m2)** | | | | **Interventricular septum thickness in diatsole (cm)** | | | | **LV posterior wall thickness (cm)** | | | | **LV internal diameter in diastole (cm)** | | | | **Mitral valve E/A Ratio** | | **Isovolumic relaxation time (ms)** | | | | **Pulmonary Vein S/D Ratio** | | | | **Global longitudinal strain** | | | | |  | | | |  | | | |  | | | |  |  |  |
| --- | --- | --- | --- | --- | --- | --- | --- | --- | --- | --- | --- | --- | --- | --- | --- | --- | --- | --- | --- | --- | --- | --- | --- | --- | --- | --- | --- | --- | --- | --- | --- | --- | --- | --- | --- | --- | --- | --- | --- | --- | --- | --- | --- | --- | --- | --- | --- | --- | --- | --- | --- | --- |
| *1* | *2* | | *1* | | *2* | | *1* | | *2* | | *1* | | *2* | | *1* | | *2* | | *1* | | *2* | | *1* | *2* | *1* | | *2* | | *1* | | *2* | | *1* | | *2* | | | |  | | | |  | | | |  | | | |  |  |
|  | 7 | |  | | 13 | |  | | 105 | |  | | 0,98 | |  | | 0,68 | |  | | 5,53 | |  | 4,50 | 115 | |  | |  | |  | | -7,9 | | -5,4 | | | |  | | | |  | | | |  | | | |  |  |
| 18 | 33 | |  | |  | | 124 | | 63 | | 0,71 | | 0,62 | | 0,99 | | 0,77 | | 6,04 | | 4,90 | | 4,00 | 1,91 | 75 | | 65 | | 0,54 | | 0,88 | | -12,7 | | -19,6 | | | |  | | | |  | | | |  | | | |  |  |
| 11 | 51 | |  | |  | | 23 | | 59 | | 1,27 | | 0,95 | | 0,91 | | 1,07 | | 1,92 | | 3,75 | | 0,99 | 1,76 | 55 | | 91 | | 0,84 | | 1,25 | | -12,8 | | -15,0 | | | |  | | | |  | | | |  | | | |  |  |
| 20 | 22 | |  | | 51 | |  | | 63 | | 1,00 | | 0,90 | | 0,97 | | 0,85 | | 3,94 | | 4,41 | | 0,80 | 0,76 | 72 | | 99 | | 1,47 | | 1,31 | | -13,1 | | -17,4 | | | |  | | | |  | | | |  | | | |  |  |
| 17 | 12 | |  | |  | | 72 | | 69 | | 0,76 | | 0,53 | | 0,85 | | 0,91 | | 4,35 | | 4,62 | | 1,26 | 1,88 | 74 | | 52 | | 1,42 | | 0,66 | | -13,2 | |  | | | |  | | | |  | | | |  | | | |  |  |
| 37 | 37 | | 63 | | 55 | | 122 | | 130 | | 1,81 | | 1,49 | | 1,28 | | 1,18 | | 3,80 | | 3,98 | | 1,49 | 1,09 | 72 | | 126 | | 1,40 | | 1,70 | | -13,9 | | -13,5 | | | |  | | | |  | | | |  | | | |  |  |
| 44 | 40 | |  | |  | | 137 | | 120 | | 1,38 | | 1,11 | | 1,15 | | 1,53 | | 4,43 | | 4,10 | |  | 2,91 |  | | 120 | |  | | 1,12 | | -13,9 | | -14,3 | | | |  | | | |  | | | |  | | | |  |  |
|  | 26 | | 51 | | 56 | | 111 | |  | | 1,42 | | 2,25 | | 1,28 | | 2,38 | | 4,39 | | 5,60 | | 0,91 | 0,94 |  | |  | | 0,64 | |  | | -14,1 | | -16,6 | | | |  | | | |  | | | |  | | | |  |  |
| 49 | 45 | |  | |  | | 79 | | 68 | | 0,98 | | 1,23 | | 1,14 | | 1,17 | | 3,52 | | 2,93 | | 0,75 | 0,75 | 117 | | 109 | |  | | 2,15 | | -14,2 | |  | | | |  | | | |  | | | |  | | | |  |  |
| 33 | 52 | | 67 | |  | |  | | 83 | | 1,34 | | 1,13 | | 1,39 | | 1,03 | | 3,79 | | 3,71 | | 0,86 | 1,66 | 123 | | 95 | | 1,71 | | 1,15 | | -14,3 | | -16,9 | | | |  | | | |  | | | |  | | | |  |  |
| 43 | 35 | |  | |  | |  | | 76 | | 0,96 | | 0,75 | | 0,90 | | 0,94 | | 5,55 | | 5,21 | | 1,44 | 1,51 | 71 | | 66 | | 0,98 | | 1,15 | | -16,1 | |  | | | |  | | | |  | | | |  | | | |  |  |
| 27 | 27 | | 66 | |  | | 77 | | 77 | | 0,92 | | 0,92 | | 0,88 | | 1,04 | | 4,84 | | 4,54 | | 0,71 | 1,12 | 83 | | 82 | | 1,94 | | 1,54 | | -16,1 | | -9,1 | | | |  | | | |  | | | |  | | | |  |  |
| 40 | 39 | |  | |  | | 54 | | 89 | | 1,05 | | 1,21 | | 1,04 | | 1,15 | | 4,58 | | 4,27 | | 1,01 | 0,86 | 84 | |  | | 1,42 | |  | | -16,9 | | -13,9 | | | |  | | | |  | | | |  | | | |  |  |
| 30 | 27 | |  | |  | |  | | 68 | | 1,02 | | 1,02 | | 0,93 | | 0,98 | | 4,45 | | 4,20 | | 1,16 | 1,11 | 83 | | 94 | | 1,85 | | 1,55 | | -17,1 | | -16,3 | | | |  | | | |  | | | |  | | | |  |  |
| 30 | 35 | | 66 | |  | | 74 | |  | | 0,81 | | 0,74 | | 0,89 | | 1,11 | | 4,43 | | 4,24 | | 2,22 | 1,27 | 45 | | 25 | | 0,59 | | 1,05 | | -17,1 | | -18,6 | | | |  | | | |  | | | |  | | | |  |  |
| 24 | 26 | | 35 | | 54 | | 80 | |  | | 0,88 | | 0,91 | | 0,89 | | 0,94 | | 4,53 | | 4,56 | | 0,73 | 0,73 | 83 | | 126 | | 1,68 | | 1,78 | | -17,6 | | -15,9 | | | |  | | | |  | | | |  | | | |  |  |
| 15 |  | | 48 | |  | | 51 | | 46 | | 0,62 | | 0,64 | | 0,91 | | 0,81 | | 3,96 | | 3,85 | | 0,89 | 0,97 | 78 | | 83 | | 1,31 | |  | | -17,7 | | -20,3 | | | |  | | | |  | | | |  | | | |  |  |
| 37 | 33 | |  | |  | | 78 | | 78 | | 0,84 | | 0,79 | | 0,77 | | 0,79 | | 5,35 | | 5,60 | | 1,97 | 2,25 | 65 | | 75 | | 1,02 | | 0,89 | | -17,7 | |  | | | |  | | | |  | | | |  | | | |  |  |
|  | 30 | |  | | 63 | | 64 | |  | | 0,83 | | 0,76 | | 0,92 | | 0,70 | | 4,55 | | 4,62 | | 1,62 | 1,23 | 92 | | 92 | |  | | 1,18 | | -18,3 | | -20,0 | | | |  | | | |  | | | |  | | | |  |  |
| 37 | 23 | |  | |  | | 118 | | 101 | | 1,25 | | 1,17 | | 1,29 | | 1,34 | | 4,73 | | 4,30 | | 1,09 | 1,16 | 105 | | 59 | | 1,34 | | 1,34 | | -18,6 | | -19,1 | | | |  | | | |  | | | |  | | | |  |  |
| 36 | 23 | | 63 | | 59 | | 82 | | 47 | |  | | 0,76 | | 0,85 | | 0,68 | | 5,17 | | 4,25 | | 3,04 | 1,72 | 77 | | 72 | | 0,69 | | 1,07 | | -18,9 | | -20,4 | | | |  | | | |  | | | |  | | | |  |  |
| 39 | 10 | | 67 | | 44 | | 59 | | 48 | | 0,65 | | 0,79 | | 0,79 | | 0,78 | | 4,74 | | 3,93 | | 1,10 | 0,66 | 69 | | 71 | | 1,58 | | 1,93 | | -19,4 | | -17,8 | | | |  | | | |  | | | |  | | | |  |  |
| 43 | 18 | |  | |  | | 65 | | 77 | | 0,85 | | 1,00 | | 0,70 | | 1,09 | | 4,80 | | 4,07 | | 0,90 | 0,72 | 72 | | 69 | |  | |  | | -19,4 | |  | | | |  | | | |  | | | |  | | | |  |  |
| 21 | 26 | |  | | 68 | | 56 | | 68 | | 0,68 | | 0,98 | | 0,87 | | 0,86 | | 4,01 | | 3,88 | | 0,93 | 1,11 | 83 | | 91 | | 0,84 | | 1,08 | | -19,6 | | -21,0 | | | |  | | | |  | | | |  | | | |  |  |
| 37 | 41 | | 68 | | 62 | | 74 | | 38 | | 0,85 | | 0,47 | | 0,90 | | 0,65 | | 4,74 | | 4,49 | | 1,40 | 1,09 | 113 | | 105 | | 1,51 | | 1,55 | | -19,9 | | -17,2 | | | |  | | | |  | | | |  | | | |  |  |
| 56 | 36 | |  | |  | | 67 | | 80 | | 0,79 | | 0,91 | | 0,83 | | 0,92 | | 4,46 | | 4,33 | | 1,26 | 2,23 | 88 | |  | | 1,12 | | 0,75 | | -20,0 | | -20,5 | | | |  | | | |  | | | |  | | | |  |  |
| 39 | 54 | |  | | 59 | | 79 | | 58 | | 0,94 | | 0,83 | | 1,02 | | 0,69 | | 3,82 | | 4,06 | | 0,95 | 1,36 | 101 | | 88 | | 2,20 | | 1,52 | | -20,7 | | -19,3 | | | |  | | | |  | | | |  | | | |  |  |
| 18 | 19 | |  | | 58 | | 84 | | 111 | | 0,97 | | 1,01 | | 0,67 | | 0,92 | | 5,85 | | 5,99 | | 1,01 | 0,95 | 80 | | 115 | | 1,58 | | 1,10 | |  | |  | | | |  | | | |  | | | |  | | | |  |  |
| 28 | 37 | |  | |  | | 34 | | 62 | | 0,60 | | 0,89 | | 0,65 | | 0,83 | | 3,72 | | 4,46 | | 1,20 | 1,17 | 59 | |  | |  | |  | |  | |  | | | |  | | | |  | | | |  | | | |  |  |
| 37 | 37 | |  | | 61 | |  | | 65 | | 0,89 | | 1,10 | | 1,05 | | 1,01 | | 4,33 | | 4,20 | | 1,20 | 0,64 | 114 | | 117 | | 1,31 | | 2,23 | |  | |  | | | |  | | | |  | | | |  | | | |  |  |
|  | |  | |  | |  | |  | |  | |  | |  | |  | |  | |  | |  | |  | |  | |  | |  | |  | |  | |  | | | |  | | | |  | | | |  | | | |  |
| Abnormal in diseased direction | | | | | |  | |  | |  | |  | |  | |  | |  | |  | |  | |  | |  | |  | |  | | low for age only | | | | |  | | | |  | | | |  | | | |  |  |  |  |
| Abnormal in opposite direction | | | | | |  | |  | |  | |  | |  | |  | |  | |  | |  | |  | |  | |  | |  | | low for both age and gender | | | | | | | | | | | | |  | | | |  |  |  |  |
|  | |  | |  | |  | |  | |  | |  | |  | |  | |  | |  | |  | |  | |  | |  | |  | |  | |  | |  | | | |  | | | |  | | | |  | | | |  |
|  | |  | |  | |  | |  | |  | |  | |  | |  | |  | |  | |  | |  | |  | |  | |  | |  | |  | |  | | | |  | | | |  | | | |  | | | |  |
